# Supra-ceramics: a molecule-driven frontier of inorganic materials

**DOI:** 10.1080/14686996.2024.2416384

**Published:** 2024-10-16

**Authors:** Kazuhiko Maeda, Teruki Motohashi, Ryo Ohtani, Kunihisa Sugimoto, Yuta Tsuji, Akihide Kuwabara, Satoshi Horike

**Affiliations:** a Department of Chemistry, School of Science, Institute of Science Tokyo, Tokyo, Japan; b Research Center for Autonomous Systems Materialogy (ASMat), Institute of Science Tokyo, Yokohama, Japan; c Department of Applied Chemistry, Faculty of Chemistry and Biochemistry, Kanagawa University, Yokohama, Japan; d Department of Chemistry, Faculty of Science, Kyushu University, Fukuoka, Japan; e Department of Chemistry, Faculty of Science and Engineering, Kindai University, Higashi-osaka, Japan; f Faculty of Engineering Sciences, Kyushu University, Fukuoka, Japan; g Nanostructures Research Laboratory, Japan Fine Ceramics Center, Nagoya, Japan; h Department of Chemistry, Graduate School of Science, Kyoto University, Kyoto, Japan

**Keywords:** Metal complexes, organic-inorganic hybrids, perovskites, metal-organic frameworks, coordination polymers, batteries, solar cells, photocatalysts, proton conductors, CO_2_ conversion

## Abstract

Discoveries and technological innovations over the past decade are transforming our understanding of the properties of ceramics, such as ‘hard’, ‘brittle’, and ‘homogeneous’. For example, inorganic crystals containing molecular anions exhibit excellent secondary battery characteristics, and the fusion of inorganic solids and molecules results in innovative catalytic functions and physical properties. Different from the conventional ceramics such as metal oxides that are formed by monatomic cations and anions, unique properties and functions can be expected in molecular-incorporated inorganic solids, due to the asymmetric and dynamic properties brought about by the constituent molecular units. We name the molecular-incorporated inorganic materials that produce innovative properties and functions as supra-ceramics. In this article, we describe various kinds of supra-ceramics from the viewpoint of synthesis, analysis and physical properties/functions for a wide range of applications.

## IMPACT STATEMENT

Recent discoveries/innovations are transforming ceramics by integrating molecular units, leading to new properties like enhanced battery performance and catalytic functions. Supra-ceramics, combining molecules and solids, promise innovative materials development.

## GRAPHICAL ABSTRACT

**Figure:**
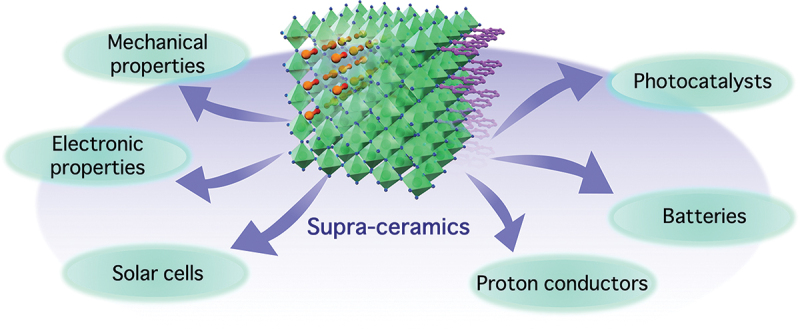

## 1. Introduction

Ceramics, represented by metal oxides, have many useful properties and functions, including high thermal stability and good transport properties, that can never be obtained by molecular materials such as organic compounds, polymers, or metal complexes. The science of metal oxides is well established from the viewpoint of cations in inorganic materials science [1]. However, because the origin of functional properties of metal oxides is defined by their coordination structure consisting of metal and oxygen atoms, the ability to develop metal oxides with desired properties/functions has been limited. Under these circumstances, mixed-anion compounds, designed around the concept of coexisting multiple monatomic anions (O^2–^, N^3–^, etc.) in a single phase, continue to evolve into a major group of materials in inorganic materials science [2–4].

Numerous properties and functions remain difficult to realize even with state-of-the-art cation/anion science in solid-state chemistry. For example, many catalytic materials, including oxides and mixed-anion compounds, have been developed as photocatalysts for the conversion of small molecules such as CO_2_ into useful chemicals, which is one of the most important issues in modern society. However, their conventional crystal-structure systems, with their electronic states and static material morphology, make dramatic advances and breakthroughs in catalysis difficult. Such challenges also exist in the field of materials design for secondary batteries and bio-ceramics, where high functionality and compatibility with other substances are required for practical applications.

To realize a breakthrough, it is essential to incorporate new degrees of freedom to propose and create a new family of ceramics. That is, a revolution in inorganic materials science requires a shift in thinking to take advantage of the structural asymmetry and dynamic properties of molecular units composed of multiple atoms.

**Figure 1.**
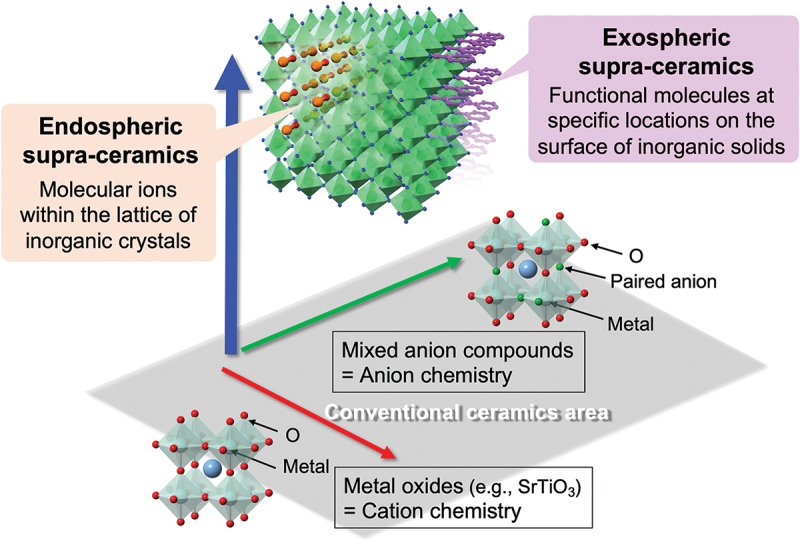
Our new concept of ‘supra-ceramics’, which provides a large space to develop materials with distinct physical properties and/or functions. The supra-ceramics are divided into two categories – endospheric and exospheric – depending on how molecular species are involved in inorganic solids.

Here, we propose a new concept of ‘supra-ceramics’, in which molecular units (e.g. molecular ions, metal complexes, or clusters) are incorporated into inorganic solids (Figure 1). The supra-ceramics can be classified into two categories – endospheric and exospheric – depending on how a molecular unit is incorporated.

### 1.1. Endospheric supra-ceramics

Endospheric supra-ceramics represent inorganic materials containing molecular-ion species [e.g., (CH_3_)_4_N^+^, (C_5_H_5_NH)^+^, OH^–^, CN^–^, SCN^–^, or (O_2_)^*n*–^] in inorganic crystal lattices. Because of the asymmetry, anisotropy, and dynamic degrees of freedom derived from molecular ionic species, new properties (e.g. low-temperature phase transitions or low volume modulus) and functions (e.g. luminescence, catalysis, electrochemical energy storage, or ionic conductivity) based on new structures and electronic states are expected. Endospheric supra-ceramics accept simple, ordinary molecular species for incorporation into inorganic materials; however, they perturb the structure and electronic state of the lattice, resulting in unique properties.

### 1.2. Exospheric supra-ceramics

Exospheric supra-ceramics are inorganic materials whose physical properties and functions are altered by functional molecules (e.g. complexes or clusters) placed at specific positions on their surface. By making maximal use of perturbations from the crystal surface or interface, researchers can create new structures, morphologies, and electronic states that inorganic materials and molecules alone do not possess. In addition to modulation of physical properties (e.g. redox change or ion – electron mixed interface conduction) as a result of the dynamic properties of the introduced molecular species, functional modification (e.g. selectivity control of catalytic reactions or rectification of transport functions) is expected. High-speed superplasticity that exhibits ‘elongation and contraction’ beyond that of metals, which cannot be envisioned within the conventional concept of ceramics, and innovative catalytic functions such as the highly efficient conversion of low-concentration CO_2_ can also be developed.

### 1.3. What can we do with supra-ceramics?

Non-spherical, small molecular species, including neutral molecules and charged ions in ceramics, would show various structural and dynamic behaviors, including isomerization, reorientation, and rotation. The presence of these molecular species results in the formation of low-symmetry crystal structures, reactive guest-accessible sites, and inherent structural dynamics, which are related to the mechanical properties and phase-transition behavior of supra-ceramics.

**Figure 2.**
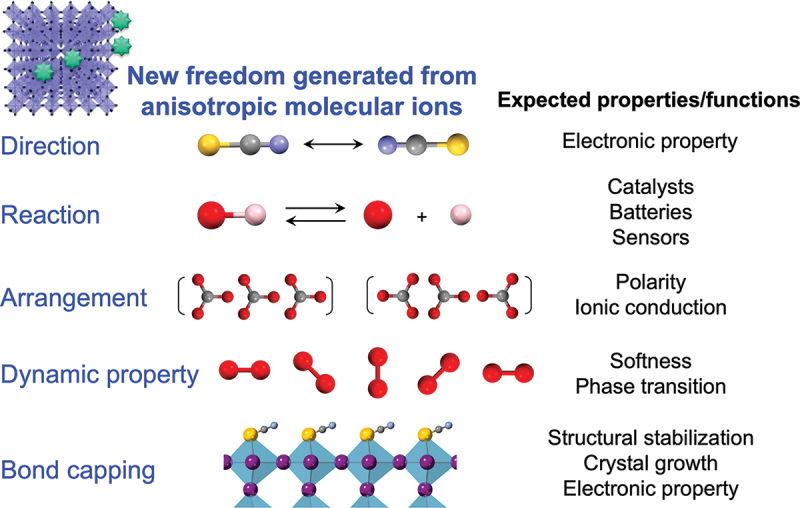
New degrees of freedom originating from non-spherical molecular species (e.g. neutral molecules and charged ions) in inorganic solids and expected properties/functions, showing the ‘core of supra-ceramics’.

Figure 2 summarizes new degrees of freedom generated in an inorganic solid as a result of the incorporation of molecular species. For example, different orientations of molecular species can change the bonding state with neighboring metal cations, leading to an alteration of the electronic properties, as discussed later. In addition, the presence of molecular anion species (e.g. OH^–^ or SH^–^) within crystals can provide reaction centers for catalysis. Other possibilities include the development of polarity by controlling the arrangement of molecular ions and the softness and phase-transition behavior of materials via their dynamic properties. Some molecular ions (e.g. SCN^−^) can function as the terminus of a chemical bond and occupy certain spaces that differ from the spaces occupied by monoatomic ions.

For endospheric-type supra-ceramics, in which molecular ionic species are incorporated inside the crystal of an inorganic material, crystallographic restrictions can limit the size of the species that can be incorporated to relatively small sizes. However, exospheric-type supra-ceramics, whose physical properties and functions are revolutionized by the placement of functional molecules at specific locations on the surface of an inorganic material, can accommodate much larger molecules such as metal complexes and aromatic molecules. Thus, exospheric-type supra-ceramics enable dramatic material design by combining the freedom of orientation, reaction, and arrangement of molecular species.

### 1.4. Early works

Although the term ‘supra-ceramics’ has not been used previously, it should be noted that certain inorganic compounds that incorporate molecular units have attracted attention in early works and some of them have become research ‘hot-spots’ in materials science. A representative example is the organic – inorganic hybrid perovskite family, which has the general formula *AMX*_3_ [*A* = CH_3_NH_3_^+^ (MA), HC(NH_2_)_2_^+^ (FA); *M* = Sn^2+^, Pb^2+^; *X* = Cl^−^, Br^−^, I^−^] [5,6]. These compounds are noteworthy because of their extraordinary physical properties, which include high mobility, a long carrier lifetime, and a low trap density, making them promising candidate materials for optoelectronic applications, including solar cells [5] and light-emitting diodes [7]. The molecular ions incorporated into the crystal structure play critical roles in the pronounced properties, as documented in recent review articles [8–10]. Notably, however, previous works on hybrid perovskites have mostly been aimed at optoelectronic applications, with materials synthesis largely limited to solution-based methods such as precipitation and solvothermal techniques. That is, much room exists for investigating molecular-incorporated inorganic compounds in terms of material functionalities and synthesis methods.

Metal – organic frameworks (MOFs) and coordination polymers (CPs) are different but important classes of materials related to supra-ceramics [11–15]. Their material chemistry has expanded rapidly, mainly as a result of the efforts of coordination chemists. Notably, these porous functionalities such as gas storage [16] and separation [17] have been demonstrated using spaces formed by low-density coordination architectures whose molecular units function as linkers between nodes such as metal ions or metal cluster units. More recently, researchers have explored further functionalities by combining porosity with characteristic electronic states of the frameworks. Electron/ion conductors [18–20] and heterogeneous catalysts [21] are applications with especially strong potential.

Organic – inorganic hybrid materials can be regarded as ‘traditional’ exospheric supra-ceramics [22,23], which include supported molecular (photo)catalysts [24–26]. In general, compared with their solid (heterogeneous) counterparts, molecular (photo)catalysts (i.e. homogeneous catalysts) show higher activity and greater selectivity for the desired product(s); however, the difficulty associated with reusing the catalysts remains. Therefore, heterogenized molecular (photo)catalysts can be regarded as good candidates for achieving both high catalytic performance and good recyclability. However, most conventional immobilized molecular catalysts simply transfer the catalytic function of the molecule in solution to the solid surface, which can sometimes result in the loss of function of the molecule by limiting its dynamic behavior on the solid surface. Placing functional molecules at targeted positions on inorganic solids is extremely difficult; however, if it becomes possible, functional amplification by molecules electronically perturbed by the solid support is expected. An interesting example is an electrochemical CO_2_ reduction catalyst consisting of a cobalt phthalocyanine immobilized on carbon nanotubes, which enabled CO_2_-to-methanol conversion; [27] however, establishing a rational strategy to control the catalytic function remains a challenge.

Examples can also be found in well known, classical materials. Since hydroxide ions are derived from water molecules, abundantly occurring (oxy)hydroxides may be categorized as endospheric supra-ceramics. These compounds have attracted interest as proton carriers in various research areas, including energy chemistry [28], catalysis [29], and electrochemistry. [30] When the proton density is low, these compounds are sometimes regarded as proton-incorporated oxides in the ceramics community. Nevertheless, it should be noted that many (oxy)hydroxides crystallize in well-ordered structures with distinct O^2–^/OH^–^ anion sites [31], highlighting the crucial role of OH^–^ anions in the construction of the crystal structure and in the emergence of functionalities.

Research on supra-ceramics aims to establish a new field of science by taking a comprehensive view of the group of materials containing molecular units (e.g. ceramics in which small molecules are incorporated), regardless of whether the materials are unknown or known. In future research on supra-ceramics, therefore, various compounds, including well-known material families, will need to be examined from a broad range of perspectives. In this review article, we present representative examples of research based on the concept of supra-ceramics, focusing on their synthesis, analysis, and properties/functions.

## 2. Synthesis

**Figure 3.**
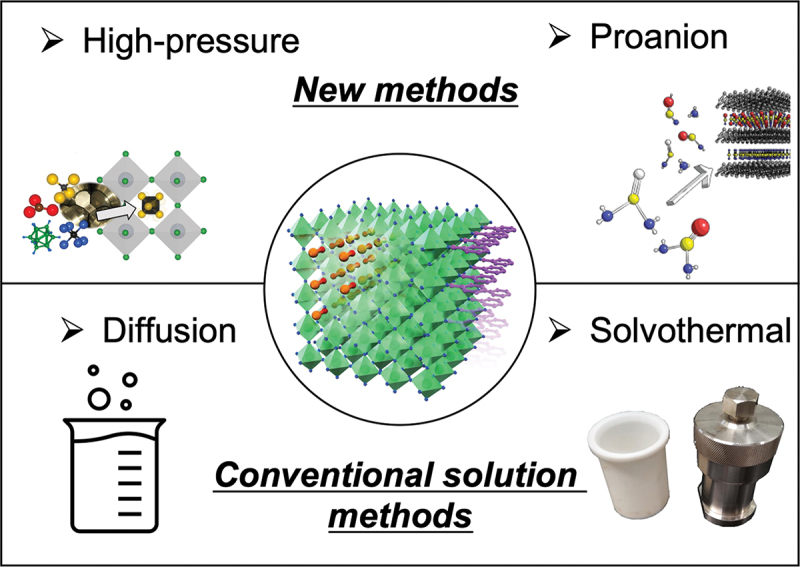
Typical and new synthesis routes for inorganic compounds that include molecular species (e.g. organic – inorganic hybrid perovskites and MOFs).

As mentioned in the preceding section, inorganic compounds that include molecular species are conventionally obtained through solution-based synthesis methods (Figure 3). The difficulty in synthesizing these compounds lies in the volatility and degradability of the starting reagents at elevated temperatures. These shortcomings hinder high-temperature synthesis routes. For the organic – inorganic hybrid perovskite family and its derivatives, several solution-based synthesis routes have proven effective. Examples include (1) solution synthesis using hydrohalic acid, (2) solution synthesis using organic solvents, (3) an antisolvent method, and (4) solvothermal synthesis [32]. For the synthesis of MOFs, routes (2) and (4) with carefully tuned temperature/reaction-time conditions are typically employed. In addition, the crystal size and morphology of MOFs are sometimes controlled by the addition of modulators to the reaction solution. Such coordination modulation methods have been established to adjust reaction equilibria, including the deprotonation of ligands, leading to fine tuning of crystal growth.

In addition to the conventional method, new schemes in which unprecedented molecules are generated during reaction processes are currently being explored to synthesize more challenging and unexplored supra-ceramics. For this purpose, it will also be necessary to understand and control the thermodynamics of the synthesis process. For example, high-pressure synthesis and the ‘proanion’ route are potential tools for developing new supra-ceramics, as discussed below. In addition, metal-carbonate frameworks were found to be generated via a wet reaction at room temperature when CO_2_ was used as a precursor in air [33].

### 2.1. High-pressure synthesis

To prevent the loss of volatile components during synthesis, the ultra-high-pressure method is highly effective. Indeed, many oxyhydrides – mixed-anion compounds containing volatile hydride anions – have been successfully synthesized using high-pressure reactions (*p* ≥3 GPa) in sealed vessels. [2] Recent studies have further demonstrated that high-pressure reactions are suitable for synthesizing molecular-incorporated inorganic compounds. Antiperovskites are notable examples of such compounds. They can be described by the formula *X*_3_*AB*, wherein the crystallographic sites are inverted compared with those in the perovskite structure. This inversion results in anions occupying both the *A* and *B* sites and cations occupying the *X* sites. Notably, the *A* site in the antiperovskite structure can accommodate larger molecular anions, as exemplified by the ZnH_4_^2−^ anion in Na_3_H(ZnH_4_) [34]. Notably, this compound cannot be synthesized using conventional solution-based methods, demonstrating the potential of the high-pressure technique to enable the preparation of novel supra-ceramics.

### 2.2. ‘Proanion’ synthesis

For the synthesis of inorganic solids that contain non-oxide anions – including (oxy)nitrides, (oxy)fluorides, and (oxy)sulfides – unconventional reagents are often used. These reagents can include ammonia (as a reactive gas), elemental sulfur, and ammonium fluoride, which serve as sources of the desired anions. Recently, research into mixed-anion compounds has paved the way for novel oxynitride synthesis methods that use nitrogen-based solids as opposed to ammonia gas [35–42]. Detailed mechanistic studies have shown that such synthesis routes invariably involve the formation of the nitride anion N^3−^ as an intermediate. Drawing parallels to terms such as ‘prochirality’, which refers to a precursor of a chiral molecule, and ‘provitamin’, denoting a parent molecule converted into a vitamin within the body, we can define a category of compounds that transform into the target anions as ‘proanions’. Notably, proanions do not actually contain the target anions, yet they transform readily on-site under typical conditions. Hence, certain nitrogen-based solids such as urea [(NH_2_)_2_CO] and carbon nitride (C_3_N_4_) act as proanions for N^3−^ ions.

**Figure 4.**
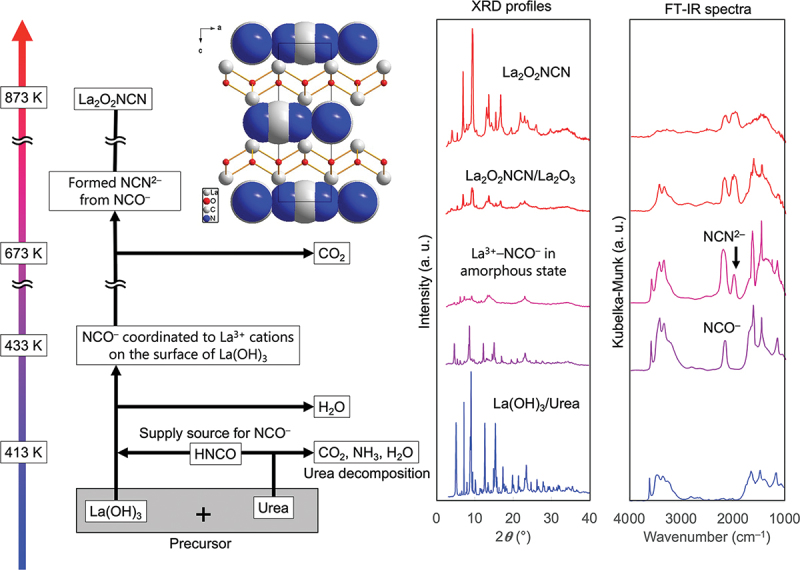
Non-ambient experiments involving X-ray diffraction profiles and FT-IR spectra have been conducted to elucidate the process for La_2_O_2_NCN synthesis using urea as a proanion.

In a recent study, Tarutani, Katagiri, and co-workers achieved facile syntheses of carbides, phosphides, sulfides, and halides starting from metal hydroxide nanoparticles mixed with various carboxylates [43]. During these synthesis processes, the added carboxylates likely act as proanions. They also demonstrated that the NCN^2−^ anion manifests as ZnNCN during the synthesis of GaN:ZnO. In addition, an oxycarbodiimide, La_2_O_2_NCN, was identified as an intermediate in the formation of LaTiO_2_N starting from La(OH)_3_ and urea [36]. These observations prompted us to use La(OH)_3_ and urea for the synthesis of phase-pure La_2_O_2_NCN. The results suggest that urea can function as a proanion of NCN^2−^, highlighting the potential for efficient carbodiimide synthesis methods that employ urea [44]. In situ Fourier transform infrared (FT-IR) spectroscopy was used to elucidate the phase formation mechanism of La_2_O_2_NCN [45]. When a mixture of La(OH)_3_ and urea was heated to 433 K under a N_2_ flow, a new absorption band was observed at *ν* ≈2150 cm^−1^, followed by the emergence of another band at *ν* ≈2000 cm^−1^ when the temperature exceeded 673 K. These bands are attributed to the cyanate NCO^−^ and carbodiimide NCN^2−^ anions, respectively. These observations suggest that isocyanic acid, HNCO, plays a critical role as an intermediate in the proanion synthesis of La_2_O_2_NCN (Figure 4). Very recently, transition metal carbodiimides, MnHf(NCN)₃ and FeHf(NCN)₃, with a structure similar to perovskites, have been reported as new compounds composed of NCN^2 −^ anions. [46] MnHf(NCN)₃ has a non-centrosymmetric structure, with chirality confirmed by second-harmonic generation measurements, and FeHf(NCN)₃ contains iron in a high-spin state. These compounds exhibit strong antiferromagnetic interactions, and MnHf(NCN)₃ has been found to possess a wide band gap and p-type semiconductor properties.

## 3. Chemical and structural analyses

**Figure 5.**
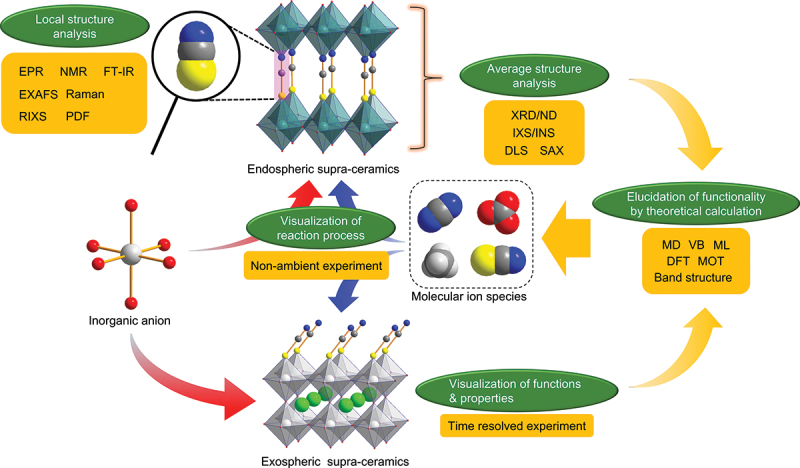
Visualization of chemical structures of supra-ceramics based on various static and dynamic advanced measurements and computational approaches.

Both endospheric and exospheric types of supra-ceramics have been studied. To develop supra-ceramics with chemical, morphological and spatial diversity from the viewpoint of structural science, conventional structural analysis methods alone are insufficient. The main methods for structural analysis include synchrotron radiation spectroscopy, neutron diffraction, transmission electron microscopy, and surface spectroscopy, which are used concertedly and to the maximum extent possible to understand the essence of supra-ceramics. First-principles density functional theory (DFT) calculations will also be used to understand the complex structure, property, and function of supra-ceramics. Constructing a suitable measurement/analysis system for macroscopic/microscopic structures and dynamic structures through the concerted use of advanced measurement techniques is critical (Figure 5).

For endospheric-type supra-ceramics, the goal is to create new physical properties and functions arising from anisotropic molecular units. In situ structural analyses based on advanced measurement methods are necessary to elucidate not only the operating mechanism of endospheric supra-ceramics but also the synthesis process. The essential role of advanced measurement methods has been exemplified by the proanion synthesis of La_2_O_2_NCN mentioned above (Figure 4). [45] In that case, even though temperature-dependent X-ray diffraction (XRD) profiles were acquired under synthesis-compatible conditions, an amorphous phase was observed and the intermediate process could not be traced. To fully understand the synthesis process of La_2_O_2_NCN, FT-IR absorption spectroscopy was necessary to analyze molecular adsorbents produced during synthesis, which revealed that CO_2_ was desorbed in the amorphous phase and that the NCN^2−^ anions was formed from NCO^−^. The as-elucidated synthesis process provides information that is useful not only for understanding intermediate states but also in the search for new supra-ceramics. In situ observations of molecular components may be more important for exospheric supra-ceramics, in which the full potential of molecular components is induced by the inorganic crystal surface, to fully understand the structure – function relationship.

**Figure 6.**
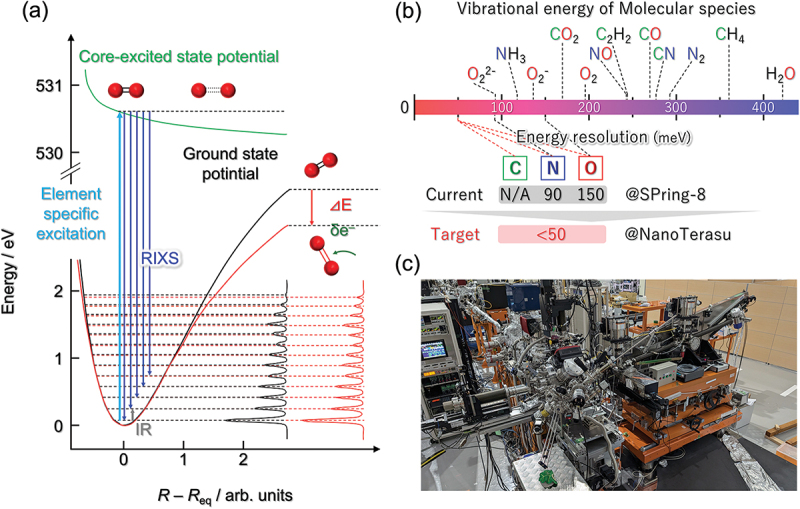
Development of RIXS local structure analysis equipment for molecular species consisting of light elements. (a) Ground-state potential energy surface obtained from RIXS vibrational spectra. (b) Vibrational energy range targeted by equipment to be developed in the near future. (c) A photograph of the RIXS instrument at NanoTerasu.

Dynamic molecular species in supra-ceramics play a key role in the development of their physiochemical properties and functions. Therefore, investigating the dynamic structure of the molecular species in solids is important. Ultra-high-resolution resonant inelastic X-ray scattering (RIXS) using soft X-rays can be used to selectively analyze the chemical states of light-element species and to calculate potential energy curves via higher-order excitation (Figure 6). Different from FT-IR and Raman spectroscopies, RIXS is a spectroscopic technique to study the resonant excitation of inner-shell electrons in materials using incident X-rays and the X-rays emitted during the relaxation of the excited state. An example of an RIXS study of supra-ceramics will be introduced in later sections.

## 4. Physical properties of endospheric supra-ceramics

In the following subsections, examples of supra-ceramics based on our concepts are introduced and discussed.

### 4.1. Drastic change in the bandgap of Cu – S – C – N (concept: direction)

**Figure 7.**
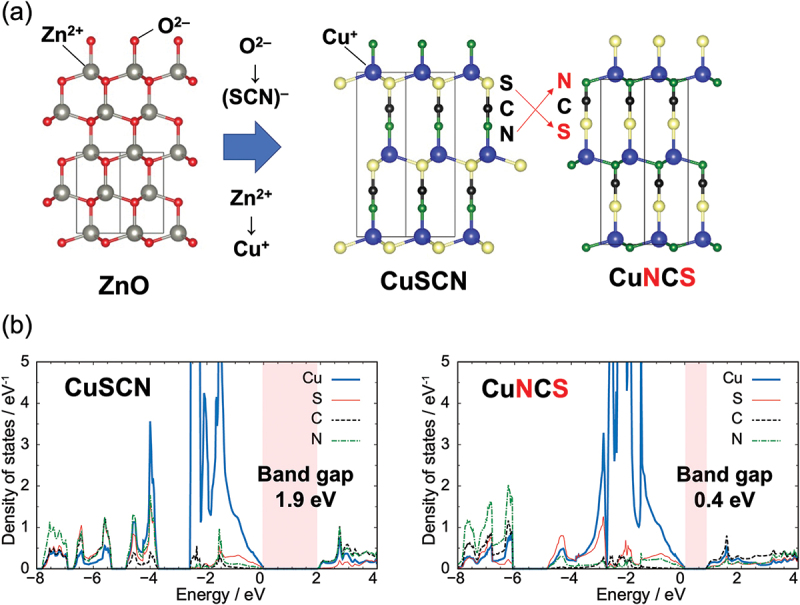
(a) Crystal structures of wurtzite-type ZnO, CuSCN, and inverse-oriented CuNCS. (b) Calculated pDOS for CuSCN and inverted CuNCS. The energy states’ valence-band top is set to 0 eV on the horizontal axis.

In addition to exhibiting anisotropic size effects not observed in monatomic ions, polyatomic ions have the characteristic of bond anisotropy. In particular, if the geometrical structure of a polyatomic ion lacks inversion symmetry, then the electronic structure and physical properties of a crystal structure in which the polyatomic ion is incorporated are expected to be changed by controlling the polyatomic ion’s orientation in the structure. We illustrate this concept using copper thiocyanate (CuSCN) as an example. CuSCN has multiple polymorphs, and β-CuSCN has the same wurtzite-type structure as zinc oxide, where Cu^+^ occupies the Zn^2+^ site of ZnO and SCN^−^ occupies the O^2−^ site. [47] The experimentally reported crystal structure of CuSCN is shown in Figure 7a. In the structure of CuSCN, Cu has tetrahedral coordination, being bonded to three sulfur atoms and one nitrogen atom. Because SCN^−^ is noncentrosymmetric, CuSCN with inverted orientation of thiocyanate ions is considered to have a structure that differs from that of the original CuSCN. This inverse-oriented structure is hereafter denoted as CuNCS. Figure 7b compares the electronic structures and partial density of states (pDOS) for CuSCN and CuNCS, as calculated by first-principles calculations. The bandgap of CuSCN is 1.9 eV, whereas that of CuNCS is 0.4 eV, or approximately one-fourth the original value. A comparison of the pDOS reveals a steep N 2p orbital peak at approximately −1.5 eV in the pDOS of CuSCN; however, in the pDOS for inverse-oriented CuNCS, this spike-like component is reduced and the N 2p orbital components are widely distributed near the valence-band top. In inverse-oriented CuNCS, one S and three N orbitals are bonded to Cu, suggesting that the N 2p orbital forms a bonding state with the Cu 3d orbital by coordinating more N atoms on the (0001) plane than in CuSCN. Such an interaction state increases the bandwidth near the valence-band top and narrows the bandgap. Unfortunately, the inverse-oriented CuNCS is 1 eV per formula unit higher in energy than the ground-state CuSCN, making the synthesis of CuNCS difficult under normal conditions. Notably, however, the orientation of polyatomic ions strongly affects the electronic properties.

### 4.2. Carbodiimides phosphors (concept: dynamic properties)

Metal carbodiimides are compounds characterized by a linear NCN^2−^ anion. This class of compounds introduces an additional degree of freedom originating from the orientation of the NCN^2−^ anion because the nitrogen atoms at both ends can bind to different cations. Consequently, carbodiimides display substantial changes in their crystal structures in response to variations in temperature and/or applied pressure. These compounds are typically synthesized either by reacting a target metal ion with a cyanamide (CN_2_H_2_) solution or through solid-state metathesis reactions involving anion exchange between a precursor carbodiimide and a halide of the desired metal. However, the synthesis of compounds with multiple metal elements, such as solid solutions or those with trace additives, are often challenging. Specifically, segregation resulting from differences in the reactivity and solubility of the constituent cations can be problematic. To address this difficulty, Masubuchi et al. developed an efficient synthesis route for complex carbodiimides that ensures cationic homogeneity. Their method involves an anion-alteration reaction starting from a complex metal carbonate, followed by high-temperature ammonolysis. [48] This technique led to the discovery of novel carbodiimides, including phosphors containing trace luminescent centers.[49,50]

**Figure 8.**
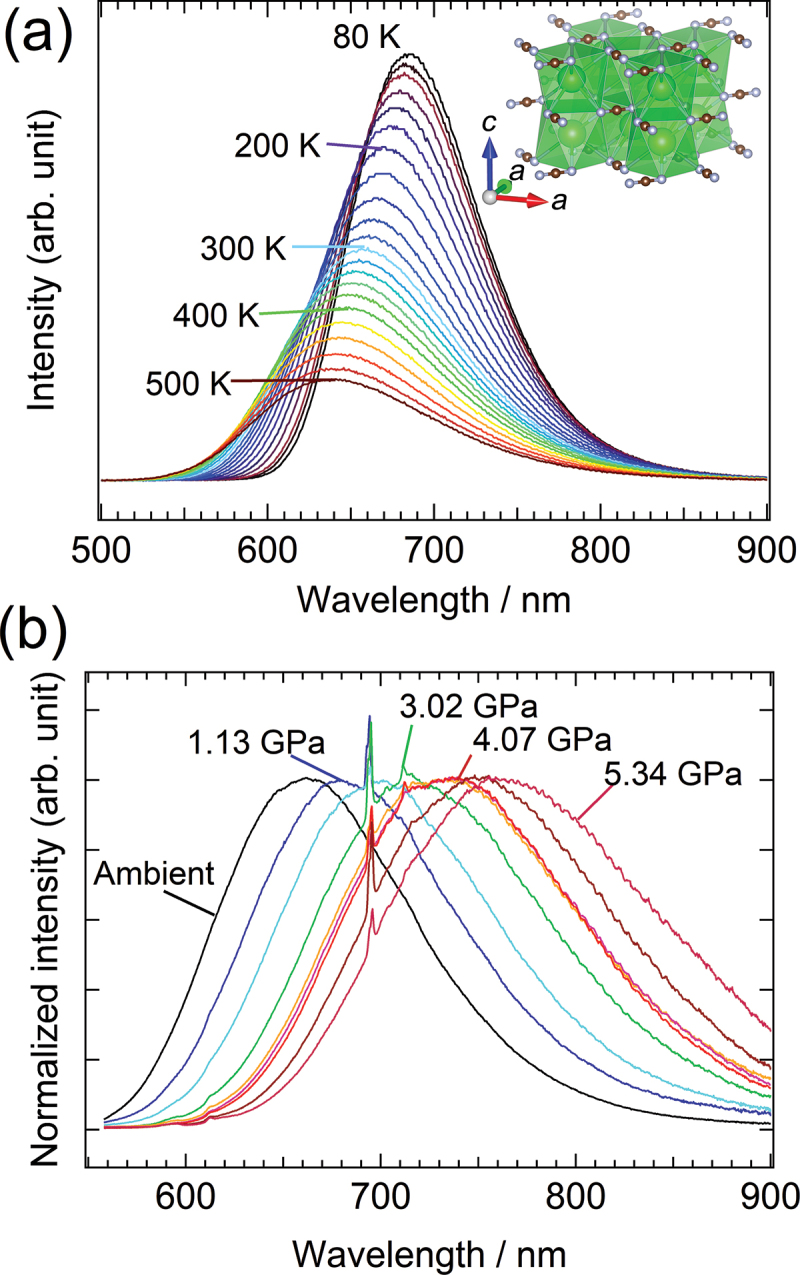
Photoluminescence spectra of Eu^2+^-doped BaCN_2_ phosphor. (a) Temperature dependence of emission spectrum under excitation at 460 nm. (b) Emission spectrum under static high pressures at room temperature.

A BaNCN:Eu^2+^ phosphor with a tetragonal structure was obtained through the ammonolysis of (Ba,Eu)CO_3_ as a precursor carbonate. Under blue-light excitation at a wavelength of 450 nm, the phosphor displays red emission. Notably, the emission wavelength exhibits a blue shift when the phosphor is heated and a red shift when the phosphor is subjected to applied pressure (Figure 8a). The resultant temperature and pressure coefficients for the emission wavelength are −0.095 nm K^−1^ and +19 nm GPa^−1^, respectively. [48,51] Notably, the pressure coefficient for the carbodiimide is approximately 50 times greater than that for ruby – a widely used material. For BaNCN, both its thermal expansion coefficient (18 × 10^−6^ K^−1^) and bulk modulus (69 GPa) differ substantially from those for the nitride Si_3_N_4_ and oxide Al_2_O_3_: they are an order of magnitude larger and smaller, respectively. The pronounced blue/red shifts in the emission wavelength are likely attributable to substantial changes in the crystal field surrounding the Eu^2+^ luminescent center. These changes arise from the crystal structure’s expansion or contraction triggered by temperature and pressure variations. These findings suggest that carbodiimides are new ceramic materials that exhibit flexible electronic states within a ‘soft lattice framework’ because of the linear NCN^2−^ anion.

### 4.3. Molecular-ion-containing soft crystal lattice that responds to chemical and physical stimuli (concept: dynamic properties)

**Figure 9.**
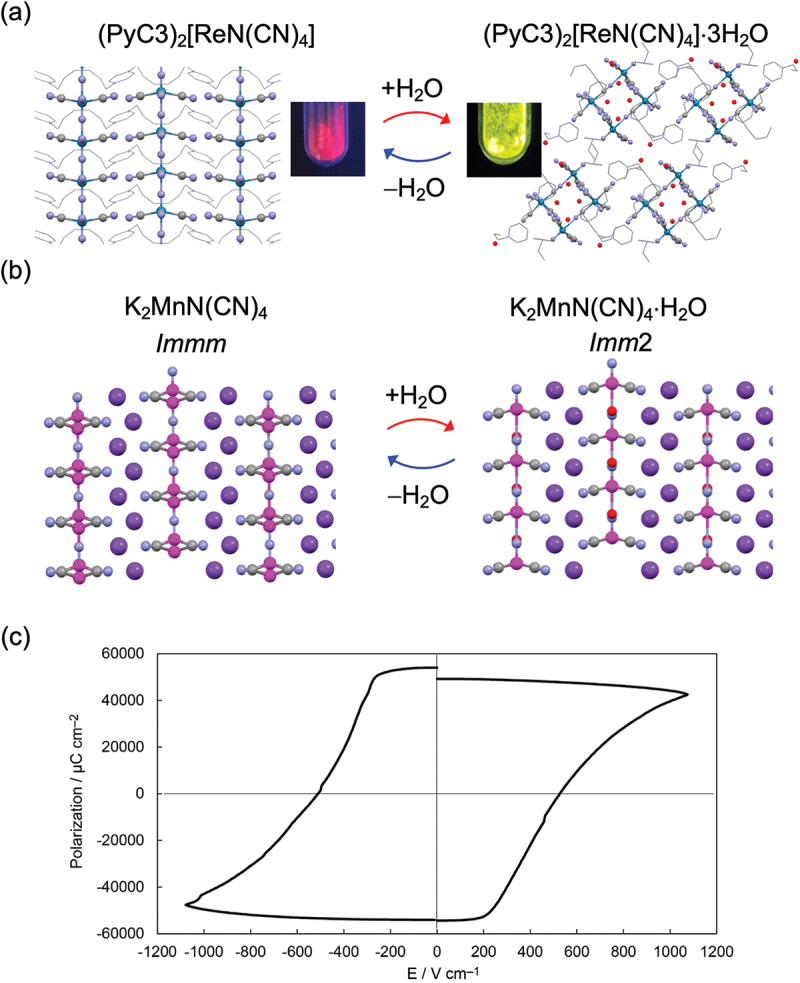
Examples of water-responsive supra-ceramics: (a) luminescence-switchable (PyC3)_2_[ReN(CN)_4_] and (b) polarity-switchable K_2_MnN(CN)_4_. (c) *P – E* hysteresis loop of K_2_MnN(CN)_4_⋅H_2_O.

Molecular ions in materials soften the dense crystalline lattice. This effect has sometimes been demonstrated by decreases in the bulk and Young’s moduli of crystals. [52] Moreover, several endospheric supra-ceramics incorporating such soft and dynamic lattices exhibit characteristic responsivities leading to reversible functionality changes in response to not only physical but also chemical stimuli such as neutral vapor.

(PyC3)_2_[ReN(CN)_4_] ([PyC3Re]; PyC3 = propylpyridinium) is an example of such a chemically responsive luminescent material incorporating a dynamic lattice (Figure 9a) [53]. [PyC3Re] consists of a one-dimensional (1D) skeleton constructed by [ReN(CN)_4_]^2−^ units assembled along the *c*-axis. PyC3^+^ is located between the chains. Remarkably, this compound adsorbs water molecules at room temperature, giving rise to a massive structural change from 1D chains to zero-dimensional cyclic clusters. The water-induced structural conversion involving ion rearrangements is reversible; that is, the clusters easily transform back to the 1D-chain form under vacuum conditions. On the basis of the structural transformation, their luminescence properties are also reversibly switched between near-infrared (739 nm) and visible (560 nm) emissions. The thermo-dynamic analysis for this system reveals a metastable liquid phase incorporating cyclic clusters in the intermediate state. Thus, the strong molecular natures in the [PyC3Re] systems could be used to demonstrate phase control of materials underpinned by a soft crystalline lattice.

Other examples are (TEA)_2_MnN(CN)_4_ (TEAMn; TEA = tetraethylammonium) and K_2_MnN(CN)_4_ (KMn), whose polarities are switchable via water adsorption/desorption at room temperature (Figure 9b). [54] A hydrated phase, TEAMnH_2_O, is composed of a noncentrosymmetric structure in the *P*4*bm* space group, whereas its dehydrated analog, TEAMn, is nonpolar in nature and exhibits *P*4/*ncc* space symmetry. The reversible polarity switching of TEAMn is caused by conformation changes of TEA^+^. That is, the presence of water can tune the geometry of molecular components, resulting in symmetry breaking of the crystal structure. Moreover, TEAMnH_2_O is a rare polar proton conductor whose proton conduction occurs along the polar axis. KMn has also demonstrated water-dependent polarity switching between space groups *Imm*2 and *Immm*. However, KMn exhibits a flexible inorganic skeleton (unlike TEAMn, whose flexible organic molecules respond to stimuli) that undergoes an order – disorder-type transition around penta-coordinate Mn centers. Importantly, the hydrated KMnH_2_O is the first example of strongly coupled functionality between ferroelectricity and proton transport; it exhibited a colossal polarization greater than 10 mC cm^−2^.

These three compounds exhibit water-vapor-induced functionality changes involving hydrogen bonding between molecular ions and water molecules as the driving force. Thus, the design of intermolecular interactions between supra-ceramic skeletons and neutral molecules is a key for the further development of materials responsive to chemical and physical stimuli.

### 4.4. Reversible color switching enabled by labile anions in inorganic solids (concept: reaction)

**Figure 10.**
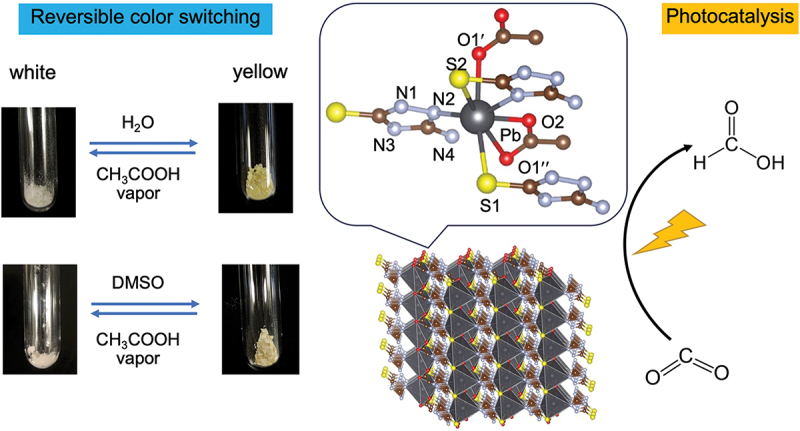
A new photofunctional Pb/S-based coordination polymer with the formula [Pb(ATAT)(OAc)]_*n*_ (ATAT = 3-amino-5-mercapto-1,2,4-triazole, OAc = acetate). Reproduced from ref. [58]. Copyright 2024, American Chemical Society.

Coordination polymers with metal – sulfur bonds (S-CPs) are an interesting group of materials that exhibit many useful properties and functions and have applications in heterogeneous photocatalysts [55], gas sensors [56], and electronic devices. [57] In particular, coordination polymers with labile ligands are expected to exhibit dynamic properties such as sensing and catalysis. A novel lead-based coordination polymer containing aminothiadiazole and acetic acid as ligands was synthesized, and its color-switching properties were found to arise from the reversible insertion and removal of acetic acid into its structure in the presence of dimethyl sulfoxide (DMSO) or H_2_O (Figure 10). [58] In addition, this coordination polymer could be used as a photocatalyst to selectively convert CO_2_ to formic acid at room temperature in the presence of 1,3-dimethyl-2-phenyl-2,3-dihydro-1 H-benzo[*d*]imidazole (BIH) under UV irradiation (*λ* < 400 nm). This work is the first report of such multifunctionality for a coordination polymer with lead – sulfur bonds.

### 4.5. Substituent position-dependent crystal structure, bandgap, and photoconductivity of lead(ii) benzenethiolate coordination polymers (concept: direction/arrangement)

**Figure 11.**
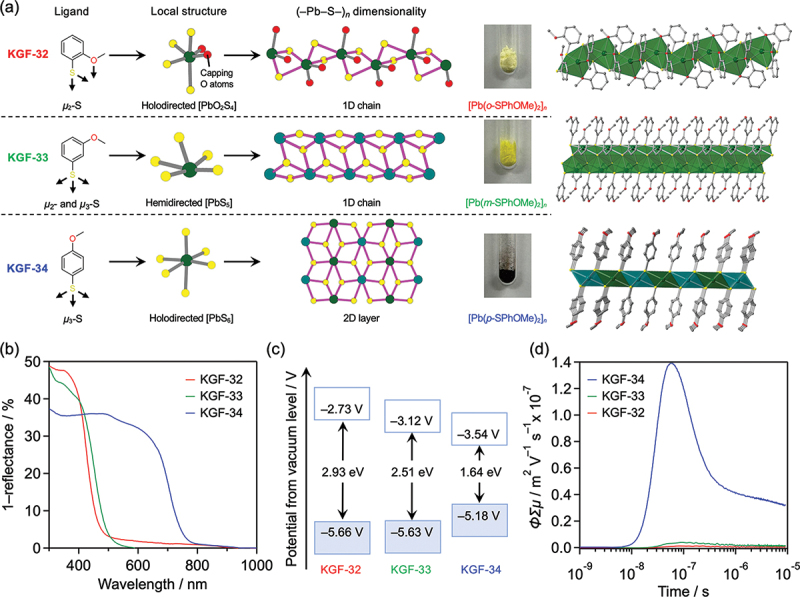
(a) Schematic of structures for [Pb(*x*-SPhOMe)_2_]_*n*_ (*x* = ortho (KGF-32), meta (KGF-33), and para (KGF-34)). From left to right: coordination modes of SPhOMe ligands, local structures around Pb(II) ions, (–Pb – S–)_*n*_ dimensionality, crystal structure, and photographs of corresponding samples. (b) UV–vis – nir spectra of KGF-32, −33 and − 34. (c) Schematic of band diagrams of KGF-32, −33, and − 34. (d) Results of TRMC measurements.

As mentioned above, the (–M – S–)_*n*_ networks formed in S-CPs are the origin of the unique properties of the coordination polymers. To investigate the semiconducting properties of S-CPs, researchers synthesized three different Pb(II) S-CPs using electron-donating methoxybenzenethiol (HSPhOMe) ligands with the methoxy substituent at different positions: [Pb(*x*-SPhOMe)_2_]_*n*_ (*x* = ortho (KGF-32), meta (KGF-33), and para (KGF-34)), which exhibit different (–Pb – S–)_*n*_ dimensionalities (Figure 11). [59] The local structures of KGF-32 and KGF-34 (ortho and para, respectively) are featured by holodirected coordination spheres composed of [PbO_2_S_4_] and [PbS_6_] octahedra, forming a 1D (–Pb – S–)_*n*_ chain and a 2D (–Pb – S–)_*n*_ layer, respectively. However, KGF-33 is composed of a hemidirected [PbS_5_] coordination sphere, which forms a 1D (–Pb – S–)_*n*_ chain. The bandgaps of KGF-32, −33, and −34 were estimated to be 2.93, 2.51, and 1.64 V, respectively, from the onset wavelengths of UV – visible diffuse reflectance spectra. Time-resolved microwave conductivity (TRMC) measurements revealed that the highest photoconductivity was obtained with KGF-34 having a 2D (–Pb – S–)_*n*_ layer, which was composed of holodirected [PbS_6_] octahedra; KGF-32 and −33 having 1D (–Pb – S–)_*n*_ chains showed moderate photoconductivity.

### 4.6. Emergent physical properties with nonspherical molecular ions (concept: arrangement)

**Figure 12.**
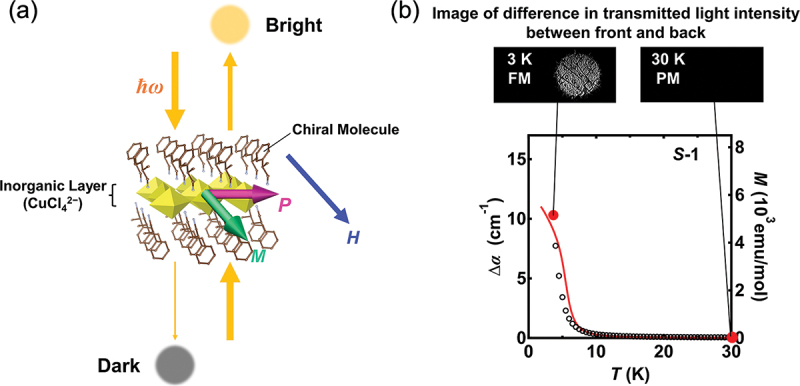
(a) Schematic of optical magneto-electric effects in (*R/S*-MPA)_2_CuCl_4_. (b) Temperature dependence of difference optical absorption coefficient. The magnetization is also plotted against temperature. The upper pictures are CCD images of the intensity difference.

The organic – inorganic hybrid perovskite (OIHP) family displays a wide structural variety, resulting in two-dimensional (2D) structures analogous to layered perovskite oxides. The 2D-OIHPs accommodate various organic molecular cations within their van der Waals gaps. Notably, bulky chiral molecules can be intercalated into the gaps, and their inherent chirality always leads to a violation of spatial inversion symmetry. [60–62] In addition, this class of compounds can induce ferromagnetism because of magnetic elements in their inorganic layers. [63] The resultant organic – inorganic composite is expected to act as a multiferroic material, simultaneously violating both spatial and time inversion symmetries. Taniguchi et al. designed and synthesized a non-inversion symmetric ferromagnet, (*R*/*S*-MPA)_2_CuCl_4_, [64] which is a 2D copper halide incorporating chiral organic ammonium cations (*R*/*S*-MPA^+^: (*R*)/(*S*)-β-methylphenethylammonium ions), as illustrated in Figure 12a.

In systems where both spatial and time inversion symmetries are broken simultaneously, the induction of the magneto-electric effect becomes permissible. Taniguchi et al. therefore attempted to induce optical magneto-electric effects in (*R*/*S*-MPA)_2_CuCl_4_ in response to a dynamic electromagnetic field (light). [64] This phenomenon is also called magneto-electric directional anisotropy (MEA), in which the optical absorption coefficient (*α*) varies when the direction of light propagation is reversed. Figure 12b displays the difference in optical absorption coefficient Δ*α* ≡ *α*(+H) − *α*(−H), together with magnetization *M*, plotted as a function of temperature. Notably, the magnitude of Δ*α* becomes substantial at temperatures below the ferromagnetic Curie temperature and correlates well with the magnetization, [64] indicating the appearance of MEA in (*R*/*S*-MPA)_2_CuCl_4_.

Ferromagnetic materials that violate spatial inversion symmetry are important because such magnets are known to exhibit novel magnetically ordered states. These states include helimagnetic and magnetic skyrmion phases, which arise from competitive interactions with ferromagnetic couplings via the Dzyaloshinskii – Moriya interaction between adjacent spins. Given that the Dzyaloshinskii – Moriya interaction includes contributions from spin – orbit couplings, Taniguchi et al. studied the Br-for-Cl-substituted compound (*R*-MPA)_2_Cu(Cl_1−*x*_Br_*x*_)_4_ with controlled spin – orbit interactions. [65] They discovered that, although the Br-free end member exhibits only a simple ferromagnetic state, the phase diagram undergoes systematic transformations with Br-for-Cl substitution, resulting in the emergence of multiple magnetically ordered phases. This result demonstrates that tuning the spin – orbit coupling, as intended, induces competition between the ferromagnetic and Dzyaloshinskii – Moriya interactions. Such noncentrosymmetric systems can lead to topological spin structures, as exemplified by the magnetic skyrmion, which has recently attracted attention. Consequently, the OIHP family holds promise as a platform for exploring novel topological materials.

### 4.7. Molecular capping inside inorganic frameworks (concept: bond capping)

**Figure 13.**
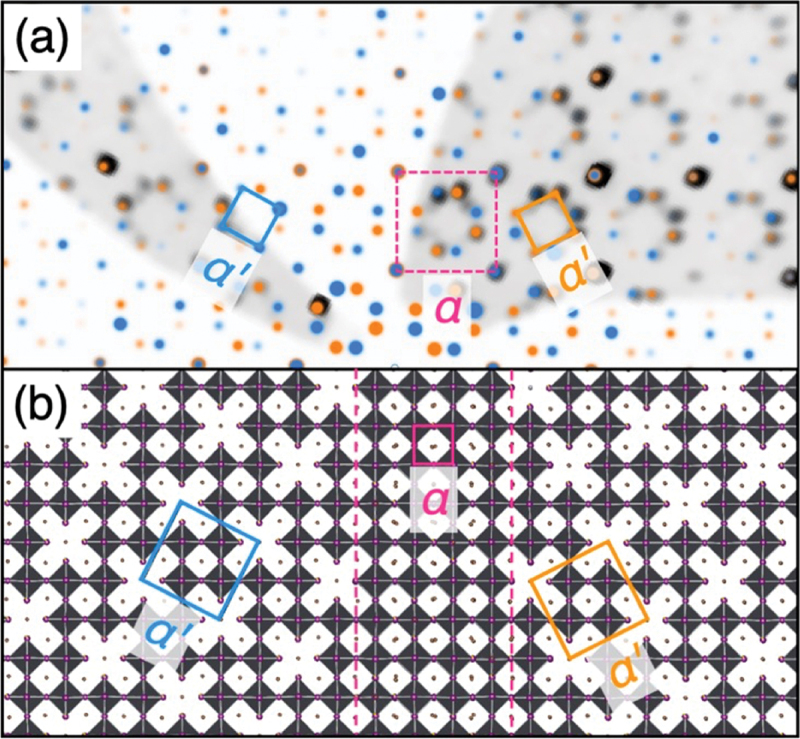
(a) Illustration of the reconstructed reciprocal lattices for the two twin components (blue and orange spots) shown on top of the unwarped, non-symmetry-averaged precession image from the diffraction data (black spots). The image is aligned with the (*hk*0)_p_ plane, with the blue twin aligned with the (210) vector and (001) plane as the upward and projection directions, and the orange twin aligned with the ($\overset{ˉ}{1}$20) vector and (00$\overset{ˉ}{1}$) plane. (b) Real-space crystal structure depictions of the vacancy-ordered superstructure at the precise orientation dictated by the twin row to illustrate the un-defective α-FAPbI_3_ present at the interface. Unit cells for the twin components of the superstructure (blue, orange) and parent structure (dashed black) are drawn on top of both reciprocal and real space images. Reproduced from ref. [66]. Copyright 2023, American Chemical Society.

The cubic-perovskite-structured α-FAPbI_3_ (FA^+^ = CH(NH_2_)_2_^+^) has an optical bandgap of 1.48 eV and is expected to be used in solar cells because of its various optoelectronic features. However, α-FAPbI_3_ is unstable; after a certain time at room temperature, it transforms to the δ phase with poor optical properties. Various studies have been conducted to control the properties of α-FAPbI_3_, including studies in which the cation and anion sites have been substituted with other ions. Recently, the introduction of the molecular anion SCN^−^ has been found to induce a characteristic structural change in FAPbI_3_. When powders of FAI (CH(NH_2_)_2_I), PbI_2_, and Pb(SCN)_2_ are reacted in the solid phase, columnar defects of [PbI]_*n*_ are formed in the three-dimensional (3D) perovskite structure. As shown in Figure 13, the extended defect forms a new structure (called the α’ phase) that forms a $\sqrt{5}$*a*_p_ ×$\sqrt{5}$*a*_p_ × *a*_p_ superlattice (*a*_p_: cell parameter for the *α* phase). [66] The I^−^ sites surrounding the columnar defects are randomly replaced with SCN^−^ ions at a rate of one in eight, and the interior of the defect is filled with FA^+^ for space and charge compensation. Such defect formation is due to the asymmetry of molecular ions of SCN^−^. SCN^−^ has an S-terminus, which forms a stable bond with Pb, and an N-terminus, which is difficult to bind to Pb. The substitution of SCN^−^ for I^−^ breaks the –(Pb – I)_*n*_– bonding chain and forms Pb–SCN–□–I– (□: Pb vacancy). This Pb vacancy aligns linearly and forms columnar defects with I, which has lost its bonding with Pb. The characteristic feature of this α’ phase is that the lattice constant per octahedron is only 0.16% different from that of defect-free α-FAPbI_3_, despite the introduction of a large concentration of columnar defects. Thus, in principle, the α and α’ phases can be connected without distortion and phases with defect patterns of different orientations can be continuously connected (see Figure 13). Heating *δ*-FAPbI_3_ with the coexisting α’ phase was also found to substantially lower the δ-to-α transition temperature. This effect may be due to a decrease in the nucleation energy as a result of the epitaxial growth of the α’ phase at the α’ interface or a result of thermodynamic stabilization by epitaxial growth. Although organic – inorganic hybrid perovskites have been extensively studied in solution synthesis, these studies have mostly been limited to thin films and single crystals. The literature contains few examples of new hybrid perovskite materials synthesized in bulk using solid-state reactions and accompanied by characterizations of their structural and phase-change behavior. The technique of molecular-ion implantation would be a powerful strategy to modulate the structure and control the properties of halide perovskites.

### 4.8. Mechanical vitrification of molecular frameworks (concept: arrangement)

**Figure 14.**
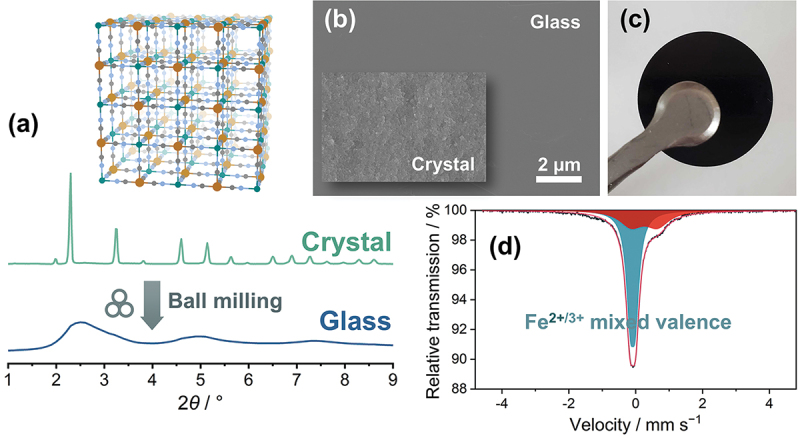
(a) Crystal structure and (B) PXRD patterns before/after mechanical milling of a CuFe PBA (K_2*x*/3_Cu^II^[Fe^II^_*x*_Fe^III^_1−*x*_(CN)_6_]_2/3_Ł_1/3_·*n*H_2_O). (b) Cross-section SEM images of crystalline and glassy CuFe PBA. (c) A photograph of the CuFe PBA glass monolith prepared by uniaxial pressing. (d) Mössbauer spectrum of the CuFe PBA at room temperature.

Prussian blue analogues (PBAs) have a three-dimensional structure in which transition-metal ions are bridged by CN^−^ ions. Most studies on PBAs have focused on the crystalline phase, and we found only a few studies on amorphous phases, especially glasses. Recently, a PBA glass was fabricated by ball-milling the corresponding crystals (Figure 14). [67] Mechanical vitrification is widely available to prepare glassy states of metals, ceramics, and even small organic molecules. Differential scanning calorimetry (DSC) measurements of a PBA composed of Fe^2+/3+^ and Cu^2+^ showed that the glass transition occurs at 333 K. X-ray total scattering measurements and subsequent pair distribution function (PDF) analysis showed that the Cu^2+^–CN – Fe^2+/3+^ connectivity remained, suggesting that a (3D) network structure was maintained in the glassy state. The CuFe PBA glass exhibits semiconducting behavior, and its conductivity is 4.1 × 10^−5^ S cm^−1^ at room temperature. This behavior is due to the mixed valence state of Fe^2+/3+^, which was confirmed by Mössbauer measurements and attributed to the preservation of the coordination bonding network. However, the Young’s modulus is substantially reduced by vitrification (12.6 GPa) and the resultant glass is highly formable, enabling the creation of high-density monoliths by uniaxial pressing. The CuFe PBA glass transforms to its original crystalline state when heated or chemically stimulated (e.g. exposed to vapors). Evaluation of the porosity using N_2_ gas adsorption isotherms acquired at 77 K shows that the vitrification – crystallization process enhances the microporosity substantially; the Brunauer – Emmett – Teller (BET) specific surface area reached 585 m^2^ g^−1^, which is almost double that of the pristine (as-prepared) crystal. This increase in specific surface area is attributed to the change in the defect structure through the vitrification – crystallization process, which creates more accessible spaces to accommodate gas molecules.

## 5. Chemical functions of endospheric supra-ceramics

### 5.1. High-capacity cathode for fluoride-ion batteries by utilizing O – O bond formation (concept: reaction)

Developing electrochemical high-energy storage systems is critically important for establishing a green and sustainable energy supply. [68] A promising candidate is fluoride-ion batteries (FIBs), which can have a far higher volumetric energy density than lithium-ion batteries. [69–76] However, the preparation of typical metal fluoride cathodes via a conversion-type reaction results in cathodes with low rate capability. Recently, layered perovskite oxides and oxyfluorides such as LaSrMnO_4_ and Sr_3_Fe_2_O_5_F_2_ have been reported to exhibit relatively high rate performance and cycle stability compared with typical metal fluoride cathodes; [77,78] however, their discharge capacities (~118 mAh g^−1^) are lower than those of cathodes typically used in lithium-ion batteries.

**Figure 15.**
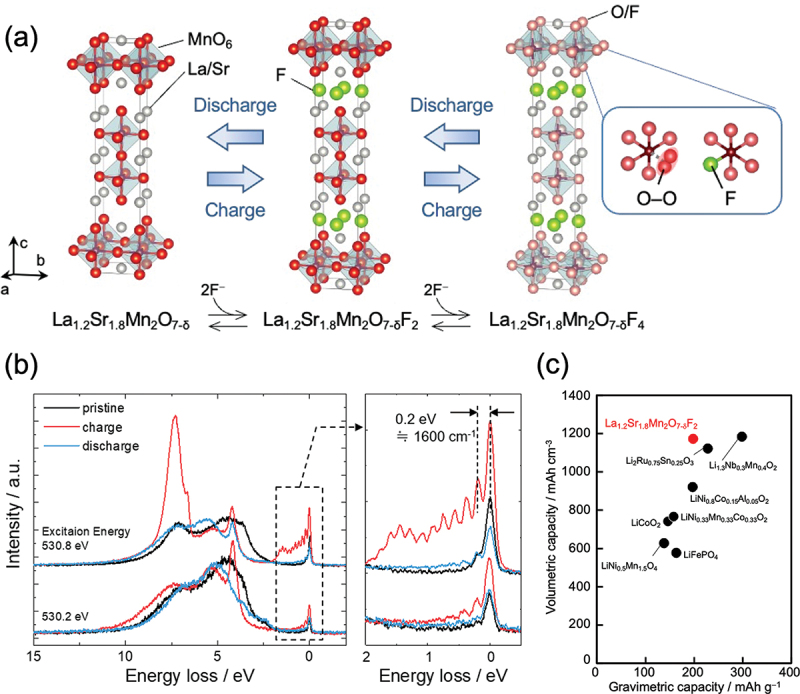
(a) Discharge/charge scheme for La_1.2_Sr_1.8_Mn_2_O_7–*δ*_F_2_ oxyfluoride. It has been unclear where O – O bonds and excess fluoride ions are located in the charged La_1.2_Sr_1.8_Mn_2_O_7–*δ*_F_2_ oxyfluoride. (b) High-resolution RIXS spectra recorded at excitation energies of 530.2 and 530.8 eV for the pristine material and the material in the charged (3.0 V) and discharged (−1.5 V) states, respectively. The vibrational frequency of ~1600 cm^−1^ indicates the presence of O – O bonds. (c) Plots of the volumetric/gravimetric capacities for La_1.2_Sr_1.8_Mn_2_O_7–*δ*_F_2_ oxyfluoride and cathode materials reported in lithium-ion batteries. Reproduced from ref. [79]. Copyright 2024, American Chemical Society.

The double-layered perovskite oxyfluoride La_1.2_Sr_1.8_Mn_2_O_7–*δ*_F_*x*_ has been shown to exhibit deintercalation/intercalation of two fluoride ions from/into rock salt slabs and further deintercalation/intercalation of excess fluoride ions from/into the perovskite layer (Figure 15a), depending on the composition (*x*). In addition to the conventional Mn redox in the range of 0 < *x* < 2, the oxyfluoride can incorporate excess fluoride ions (2 < *x* < 4) into the perovskite blocks by forming O – O bonds (i.e. anion redox, Figure 15b), which leads to a reversible capacity of 200 mAh g^−1^. [79] This value is comparable to or higher than those reported for lithium-ion batteries (Figure 15c). [80–83] It is clear that the formation of molecule-like O_2_ species in the oxyfluoride perovskite, as revealed by the RIXS technique, is clearly the key to realizing capacity improvements, but it is not obvious in the conventional *AB*O_3_-type perovskites with a close-packed structure. This research has also indicated that, for the synthesis of oxyfluorides, electrochemical conditions may provide new materials that exhibit outstanding functions that are unattainable with the corresponding materials synthesized using ordinary methods.

### 5.2. CO_2_ reduction using molecular anions (concept: reaction)

The catalytic conversion of CO_2_ into value-added chemicals has become a research topic of great importance in recent years because it may provide a solution to both global warming and the global energy shortage. Among various methods and schemes proposed thus far, photocatalytic CO_2_ reduction is promising because, in principle, it can operate at room temperature and under ambient pressure. [25,84–86] Reduction of CO_2_ by an electron requires a very high potential, and the produced CO_2_^•–^ is unstable, making product selectivity difficult to control. However, CO_2_ reduction involving proton-coupled electron transfer (PCET) substantially lowers the potential required to drive the reaction. [25] Therefore, catalysts that efficiently reduce CO_2_ into a desired product through PCET are highly desirable.

Alpha-iron(III) oxyhydroxide (α-FeOOH; goethite), the main component of iron rust, has been shown to act as a good CO_2_ conversion catalyst to give formic acid with 80–90% selectivity when used in combination with a Ru(II) redox photosensitizer. [87] In this system, the presence of large amounts of hydroxide (OH^−^) anions in the crystals can create catalytically active sites, and the catalytic reaction is thought to proceed efficiently. In this way, even a simple molecular ion species consisting of two or three atoms connected by covalent bonds can produce dramatic changes in physical properties and functions.

**Figure 16.**
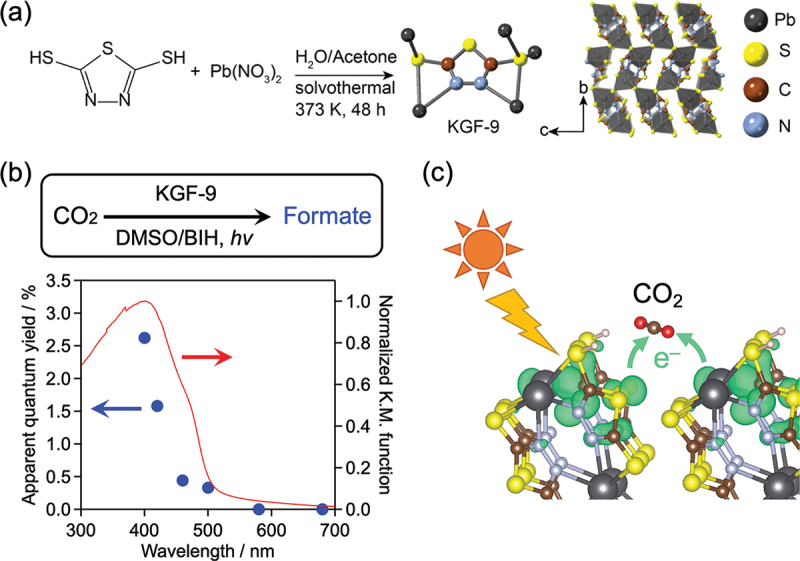
(a) Synthesis scheme and crystal structure of KGF-9. (b) UV – visible diffuse-reflectance spectrum of KGF-9 and an action spectrum for CO_2_-to-formate conversion. The reactions were conducted under monochromatized light irradiation in a DMSO solution of BIH as a sacrificial electron donor. (c) Schematic of CO_2_ reduction over the KGF-9 surface. Reproduced from refs. [89,90]. Copyright 2022 and 2024, American Chemical Society.

[Pb(tadt)]_*n*_ (named KGF-9), composed of lead ion and 1,3,4-thiadiazole-2,5-dithiol (H_2_tadt: 1,3,4-thiadiazole-2,5-dithiol), [88] is a photoconductive CP with a Pb – S bond; it exhibits semiconductor-like steep absorption in the visible-light region. Figure 16a shows the synthesis scheme, crystal structure, and optical absorption properties of KGF-9. KGF-9, which was originally synthesized by a solvothermal reaction using Pb(NO_3_)_2_ and H_2_tadt in a water/acetone mixture at 373 K for 48 h, is a CP with a 2D sheet structure of Pb and S, where the sheet structure is bridged by a five-membered ring. [88] The bottom of the conduction band of KGF-9 is more negative than the potential required to reduce CO_2_ to formic acid, CO, methanol, or methane, making it a candidate photocatalyst for CO_2_ reduction. It has also been reported as a stable CP in water and air and exhibits some thermal tolerance, not decomposing until ~ 600 K.

Selective reduction of CO_2_ into HCOOH over the KGF-9 was demonstrated under bandgap photoexcitation of the material in the presence of a sacrificial electron donor, resulting in an apparent quantum yield (AQY) of 2.6% at 400 nm (Figure 16b), which was the highest value reported for a single-component CO_2_-to-HCOOH conversion photocatalyst that does not contain a precious or rare metal. [89] Theoretical calculations indicated that – SH groups attached to the tadt ligand on the surface of KGF-9 play an important role in converting CO_2_ into HCOOH. [90] KGF-9 possessing a convex and concave surface has been suggested to be suitable for adsorption and hydrogenation of CO_2_, and the electronic features of the surface – SH groups may be suitable for electron injection into CO_2_ (Figure 16c). A tin(II)-based MOF with a trithiocyanuric acid linker having a ~2.5 eV bandgap, named KGF-10, [91] enabled CO_2_ photoreduction with an AQY of 9.8% at 400 nm.[92]

KGF-9 differs from conventional porous MOFs/CPs in that its structure has no pores. Therefore, the specific surface area of the original KGF-9 is extremely small (<1 m^2^ g^−1^). If a high specific surface area can be achieved in the future by exploring synthetic methods, it is expected to lead to even higher activity. KGF-9 having a larger specific surface area might be obtained by applying new synthesis routes. [93] For example, the use of lead(II) acetate as an alternative precursor promotes the deprotonation of the H_2_tadt ligand, resulting in rapid production of KGF-9 with a reduced feature size and an increased specific surface area (~10 m^2^ g^−1^) even at room temperature for 60 min. The as-synthesized new KGF-9 exhibited a higher AQY for HCOOH generation (5.9% at 400 nm), which is attributed to the presence of long-lived charge carriers, as revealed by time-resolved microwave conductivity measurements.

## 6. Physical properties and chemical functions of exospheric supra-ceramics

### 6.1. Electrostatic and dimensional control toward low-melting supra-ceramics

Metal phosphates exhibit various properties; for example, Ag_3_PO_4_ is known to function as a photocatalyst with high oxidation ability under visible light. [94] The insertion of molecular units into the structures of metal phosphates can substantially reduce the phosphates’ melting points. For example, the melting point of Zn_3_(PO_4_)_2_ is 1173 K; however, when an imidazole that forms hydrogen bonds is introduced into its structure, a low-dimensional crystal structure consisting of a proton-rich phosphate unit and Zn^2+^ can be synthesized. [Zn(HPO_4_)(H_2_PO_4_)_2_] (H_2_Im) (H_2_Im = monoprotonated imidazole) has a 1D chain structure composed of Zn^2+^ cations and H_2_PO_4_^−^ and HPO_4_^2−^ anions, with imidazoles intercalated between the chains. Because of the low dimensionality and high ionicity of the crystal structure, the crystal melts at 442 K to form a colorless melt. When this melt is cooled, it transforms into a glass (called Zn-imidazole glass), which exhibits high proton conductivity even under non-humidified atmospheres. According to the temperature windows for this solid-to-liquid phase transition, various molecular and/or ionic doping schemes are possible. Iron-porphyrin complexes show good photocatalytic properties for CO_2_ conversion. Small amounts of the iron-porphyrin complex were doped into Zn-imidazole glass and stabilized, and the composite film acted as a solid photocatalyst with highly selective CO generation.[95]

**Figure 17.**
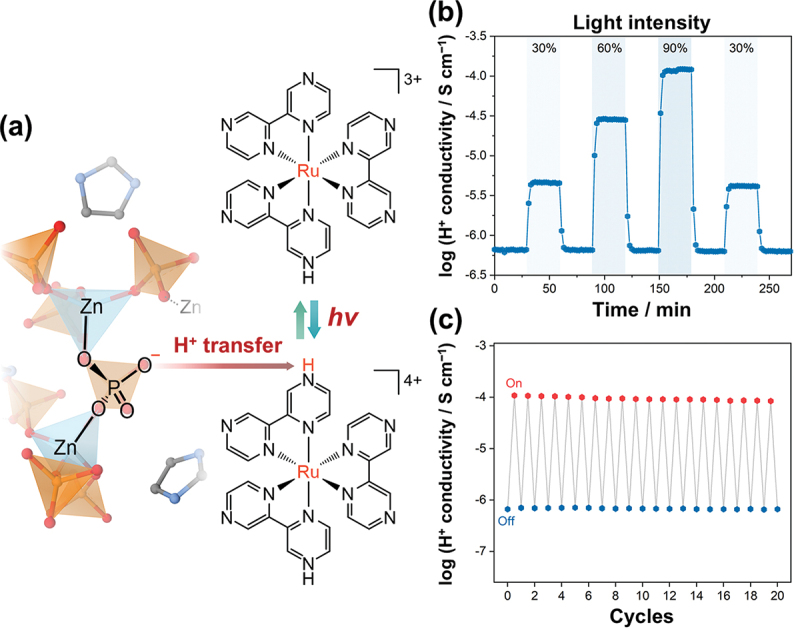
(a) Schematic of doping of tris(bipyrazine)ruthenium(ii) complex into Zn-imidazole glass [Zn(HPO_4_)(H_2_PO_4_)_2_] to promote H^+^ transfer. (b) Photoexcited H^+^ conductivity of the composite at 303 K. Blue highlights demonstrate light irradiation with different intensities. (c) Cycling stability of the photoexcited H^+^ conductivity.

A small amount of tris (bipyrazine)ruthenium(II) complex, which exhibits photoexcitation behavior, was also doped similarly into the Zn-imidazole glass (Figure 17). The doped transparent glass demonstrated rapid and sensitive switching behavior of the proton conductivity upon light irradiation, with high cyclability. [96] Proton transfer from the Zn-imidazole glass to the doped Ru complex increases the amount of proton defects, and the photoresponsiveness is manifested throughout the bulk glass. [96] Although the main structures are composed of metal phosphates, the incorporation of molecules into the crystal structure influences the thermal properties, including the melting and glass-formation behavior. The working-temperature regime below 473 K enables the use of various molecular/ionic dopants, and functional composites, including catalysts and photoactive conductors, have been fabricated.

### 6.2. Construction of MOFs on inorganic crystal surfaces for exospheric supra-ceramics

**Figure 18.**
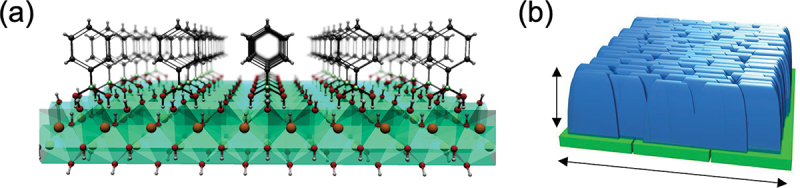
(a) Image of regular arrangements of organic molecules on an inorganic crystal surface. (b) Image of the anisotropic growth of MOF crystals on the surface.

Using the crystal surface to control the arrangement of organic molecules based on the characteristic regularity reflecting the inorganic crystal lattice, a molecular architecture can be derived whose functionality differs from that of the bulk. This idea can be further expanded into framework construction combined with coordination-driven assemblies useful for MOF architectures, leading to the creation of functional exospheric supra-ceramic devices. Takahashi et al. used this scheme with oriented Cu(OH)_2_ nanotubes or nanobelt thin films (Figure 18). [97] They demonstrated the anisotropic formation of MOF crystals consisting of 1,4-benzenedicarboxylic acid, where the *a*- and *b*-axes of the MOF crystal were aligned along with the *c*- and *a*-axes of the Cu(OH)_2_ substrate, respectively. In addition, they demonstrated that such exospheric supra-ceramic thin films exhibited anisotropic electrical conductivity that is difficult to achieve through functional design of simple bulk systems.[98]

### 6.3. Improved CO_2_ reduction by assembling a binuclear Ru(II)–Re(I) complex on TiO_2_ nanocrystals with polymeric carbon nitride

**Figure 19.**
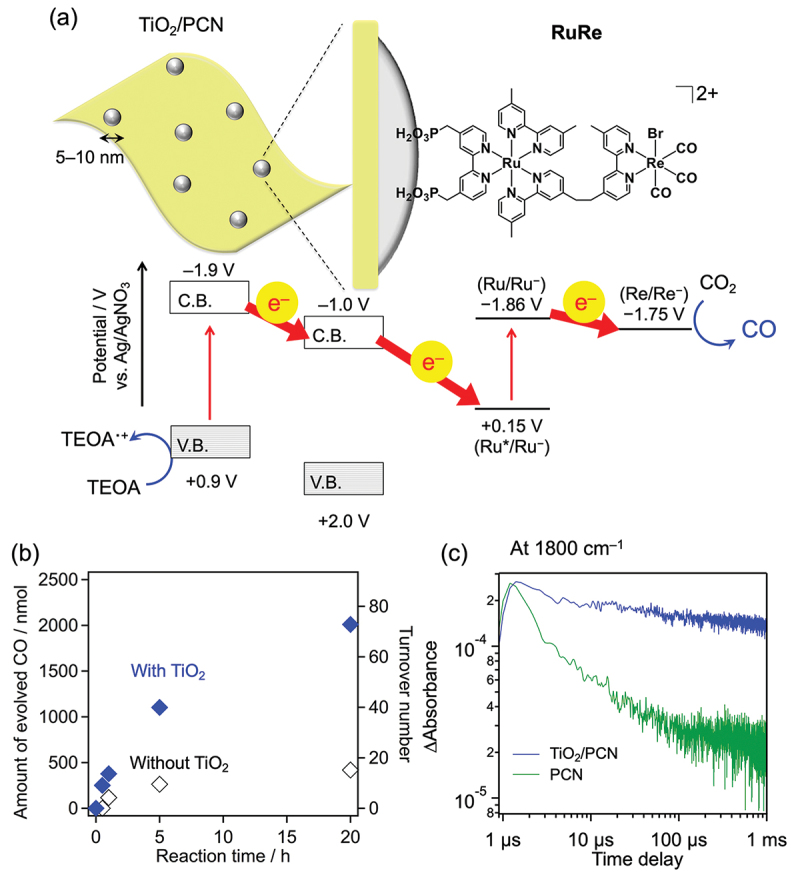
(a) Schematic of the **RuRe**-adsorbed TiO_2_/PCN hybrid photocatalyst. (b) Effect of TiO_2_ modification (27 wt%) on CO_2_ reduction by **RuRe** (6.9 μmol g^−1^) adsorbed PCN (*λ* > 400 nm), where the reduction was performed in an acetonitrile/triethanolamine (TEOA) mixture. Here, TEOA acts as a sacrificial reductant that consumes holes generated in the valence band of PCN. (c) Time-dependent transient absorption signals of PCN and TiO_2_ (27 wt%)/PCN observed at 1800 cm^−1^. Reproduced from ref. [100]. Copyright 2017, American Chemical Society.

Hybrid photocatalysts consisting of a binuclear Ru(II) – Re(I) complex (**RuRe**) combined with polymeric carbon nitride (PCN) can selectively reduce CO_2_ to CO under visible light. [99] However, the turnover number (TON) of the **RuRe**-based photocatalyst, which indicates its durability, is low; improvements in the performance of the hybrid photocatalyst are urgently needed. We attempted to improve the performance by loading 5–10 nm TiO_2_ nanoparticles onto PCN nanosheets with the objective of promoting charge separation of carriers generated in PCN by visible-light excitation and forming strong bonds with the anchor ligand (methylphosphonate group) of **RuRe** (Figure 19a). The TiO_2_-loaded PCN, adsorbed with **RuRe**, was used as a photocatalyst. As shown in Figure 19b, the CO formation rate was approximately four times higher than that of **RuRe**/PCN. [100] The TON_CO_ reached 73, which is approximately four times higher than that for conventional PCN. The TiO_2_-modified PCN showed a substantial increase in the lifetime of excited electrons (Figure 19c). [100,101] In addition, the desorption of **RuRe**, which is directly related to the low durability of the **RuRe**/PCN, was substantially suppressed by the TiO_2_ modification. These results explain the high CO production rate and high TON. That is, the function of the metal complex photocatalyst as a single molecule was shown to be greatly improved by binding the metal complex to the TiO_2_ solid surface. Such functional modification of molecular (photo)catalysts is expected to be applicable to other highly challenging small-molecule conversion reactions.

### 6.4. Theoretical chemistry to illuminate the molecular perturbations induced by inorganic solid surfaces

The advent of exospheric supra-ceramics is a pioneering endeavor aimed at fundamentally altering the physical attributes and functionalities of inorganic materials through the strategic incorporation of functional molecules onto their surfaces. Crucial to the realization of such innovation is the intricate electronic interplay between solid inorganic surfaces and molecular entities, encompassing organic molecules, metal clusters, and metal complexes. Central to this pursuit is the meticulous amplification of perturbations originating from solid surfaces, thereby engendering novel structural configurations, morphological manifestations, and electronic states beyond the realm of standalone inorganic materials or isolated molecules.

Within this domain, theoreticians strive to distill the quintessence from the intricate architectures of exospheric supra-ceramics, employing first-principles calculations to scrutinize these highly complex systems. By eschewing conventional force-field calculations in favor of first-principles methodologies, researchers have developed a comprehensive understanding of the nature of perturbations inflicted upon molecular entities by the surface, as discerned through an electronic perspective. In this section, we showcase two illustrative examples in which first-principles simulations serve as pivotal tools for revealing the underlying mechanisms governing the reactivity and physical attributes of exospheric supra-ceramics.

**Figure 20.**
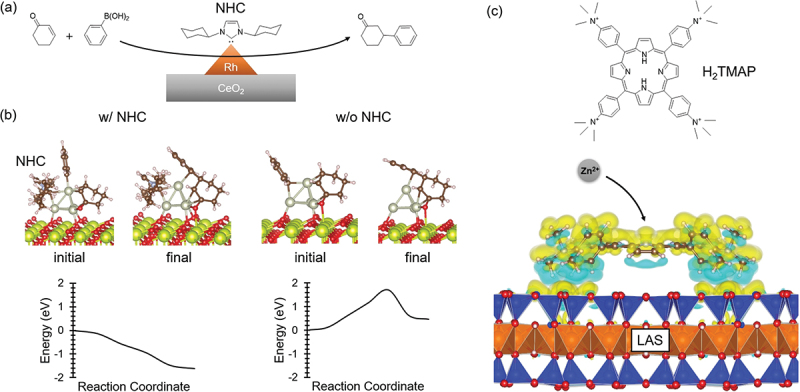
(a) Schematic of 1,4-arylation of cyclohexenone with phenylboronic acid on a ceria catalyst supporting a Rh nanocluster coordinated by NHC. (b) Initial and final state structures of the C – C bond formation step calculated for the catalytic reaction with and without NHC, and the corresponding energy diagrams. (c) Charge density difference calculated for H_2_TMAP adsorbed onto the LAS surface. Yellow and light-blue regions represent charge accumulation and depletion, respectively. VESTA was used to draw these structures. [103].

The inaugural case involves coordination-induced 1,4-arylation catalytic activity of a distinguished ceria catalyst, denoted as r-Cr_0.19_Rh_0.06_CeO_*z*_, featuring N-heterocyclic carbene (NHC) coordination. [102] Undoubtedly, this catalyst merits recognition as an exemplar of exospheric supra-ceramics. Guided by spectroscopic insights, a catalytic framework was postulated, wherein the NHC-coordinated Rh nanocluster serves as the catalytic active site (Figure 20a). Despite the anticipated facilitation of catalytic reactivity because of NHC’s pronounced electron-donating nature, its actual role proved to be more nuanced.

Contrary to the expectation, meticulous first-principles simulations shed light on the intricacies of the reaction mechanism (Figure 20b). Herein lies a theoretical revelation: NHC exerts control over the adsorption locations of the phenyl group on the Rh nanocluster, fostering the formation of a C – C bond between the phenyl group and cyclohexenone. Remarkably, it is not the electronic attributes of NHC but rather its structural influence that emerges as the driving force behind this phenomenon. NHC deftly engineers the arrangement of adsorption sites, orchestrating structural perturbations on inorganic solid surfaces to bring reactant molecules into close proximity, thereby facilitating a barrier-free bond-forming reaction. This seminal discovery advances the frontiers of catalyst design, elucidating the potential of coordination-induced catalysis within the intricate milieu of heterogeneous catalytic systems.

The subsequent illustration involves the expedited progression of an electrophilic metal coordination reaction facilitated by a layered aluminosilicate (LAS) surface complexed with trimethylammoniophenyl porphyrin (H_2_TMAP). [104] This configuration also merits recognition as an exospheric supra-ceramic. Notably, the LAS surface bears a negative charge, whereas H_2_TMAP features four cationic sites (Figure 20c) that serve as ligands for adsorption onto and the formation of strong bonds with the LAS surface. Notably, within the LAS and H_2_TMAP complex, one facet of H_2_TMAP is concealed by the surface, reducing the likelihood of collision between a metal ion and H_2_TMAP. However, intriguingly, the complexation yields an augmentation in the rate constant for the electrophilic metal coordination reaction.

First-principles calculations were carried out to elucidate this phenomenon, and the alteration in charge density accompanying the adsorption of H_2_TMAP was meticulously evaluated via charge-density difference analysis. The findings (Figure 20c) show that the interaction between H_2_TMAP and the LAS surface induces an accumulation of charge on the side opposite the LAS surface; given the negatively charged nature of the LAS surface, electrostatic repulsion forces are speculated to push the electron cloud of H_2_TMAP outward. This behavior exemplifies how the electronic perturbation originating from the inorganic solid surface enhances the functionality of the molecular unit – a noteworthy demonstration of synergy between the two realms.

### 6.5. An efficient H_2_ evolution photocatalyst constructed through interlayer modification of layered oxynitride K_2_LaTa_2_O_6_N

**Figure 21.**
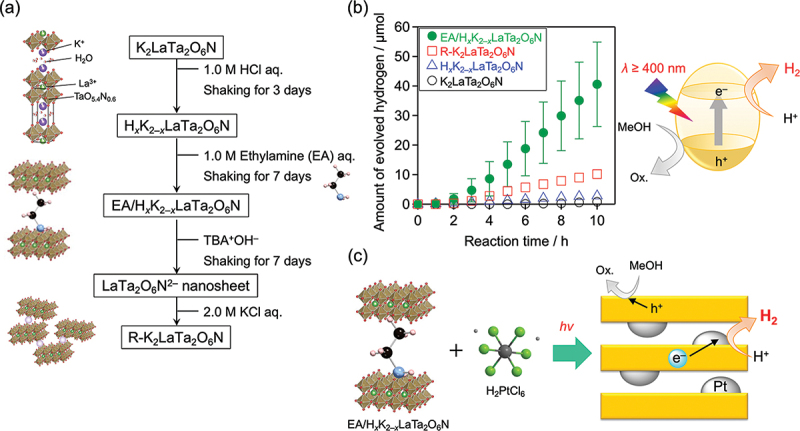
(a) Procedures used for the interlayer modification of K_2_LaTa_2_O_6_N. (b) H_2_ evolution over time using the K_2_LaTa_2_O_6_N-based materials under visible light (*λ* > 400 nm). Reaction conditions: catalyst, 50 mg (Pt photodeposited in situ); reactant solution, aqueous methanol (10 vol%, 140 mL); light source, 300 W xenon lamp with a cutoff filter. (c) Schematic of the reaction mechanism by which EA-intercalated K_2−*x*_H_*x*_LaTa_2_O_6_N reacts with H_2_PtCl_6_ to form Pt nanoparticles under illumination. Reproduced from ref. [108]. Copyright 2023, royal society of chemistry.

Semiconducting layered materials are known to function as good photocatalysts because of their unique properties, which include the ability to intercalate reactant molecules and the occurrence of anisotropic charge transport in the 2D sheets. [105,106] K_2_LaTa_2_O_6_N is a Ruddlesden – Popper-type layered perovskite oxynitride that consists of 2D LaTa_2_O_6_N^2−^ sheets interleaved with K^+^ cations for charge compensation. [107] Under visible light, K_2_LaTa_2_O_6_N modified with a metal cocatalyst such as Pt or Ir shows photocatalytic activity toward H_2_ evolution from water in the presence of a suitable electron donor (e.g. methanol or iodide). Because of the ion-exchangeability, K_2_LaTa_2_O_6_N undergoes proton-exchange in aqueous solution and exfoliation into single-layer nanosheets by reaction with bulky base molecules (Figure 21a). The H_2_ evolution activity from aqueous methanol was increased by a factor of ~ 60 when the interlayer spacing of the host K_2_LaTa_2_O_6_N was increased through H^+^/K^+^ exchange and subsequent ethylamine (EA) intercalation (Figure 21b), resulting in an AQY of 2.0% at 420 nm. In the illuminated aqueous solution, the EA-intercalated material could react with H_2_PtCl_6_, the precursor of the Pt cocatalyst, in the interlayer nanospace of K_2−*x*_H_*x*_LaTa_2_O_6_N(Figure 21c). Electrons photogenerated in the 2D LaTa_2_O_6_N^2−^ sheets reduce H_2_PtCl_6_ to Pt, producing Pt-intercalated K_2−*x*_H_*x*_LaTa_2_O_6_N, which exhibits good photocatalytic activity. However, exfoliation of the EA-intercalated material and reassembly into nanosheet aggregates resulted in a decrease in activity compared with that of the EA-intercalated samples. These results indicate that the intercalation of EA provided interlayer nanospaces suitable for Pt cocatalyst loading and promoted the photocatalytic H_2_ evolution reaction, whereas the reassembled nanosheets did not provide an interlayer space suitable for Pt loading.

### 6.6. Improved (photo)electrochemical water splitting by electron-spin control with chiral molecules

In (photo)electrochemical water oxidation to form O_2_ molecules having a triplet ground state, a certain overpotential is experimentally known to be required. This requirement can be explained in terms of a restriction on the spin of electrons involved in the generation of O_2_. In uncontrolled spin alignment, hydrogen peroxide (H_2_O_2_) is formed, contributing to the high overpotential. Photoanodes modified with chiral molecules (e.g. Zn-porphyrin and triarylamine) showed an enhanced anodic photocurrent density compared with photoanodes modified with achiral molecules. [109] In addition, spectrophotometric titration experiments showed that electrodes modified with achiral molecules produced a substantial amount of H_2_O_2_, whereas those with chiral ones did not. It was proposed that, when electrons conductive to the anode are spin specific, the spins of the two electrons are aligned parallel to each other, resulting in an interaction with two ^•^OH radicals on a triplet surface, inhibiting the formation of H_2_O_2_ and facilitating O_2_ evolution in the triplet ground state. According to the same concept, other chiral anodes based on Fe_3_O_4_ [110], CuO [111], CoO_*x*_ [112], and MoS_2_ [113] have been developed for improved electrochemical water oxidation.

### 6.7. Surface passivation for hybrid perovskite solar cells

As mentioned earlier, OIHPs have stimulated great interest for application in solar cells; [5] however, their intrinsic weakness in contact with moisture under illumination hinders their large-scale application. [114] Therefore, improving the durability of hybrid perovskites is important.

**Figure 22.**
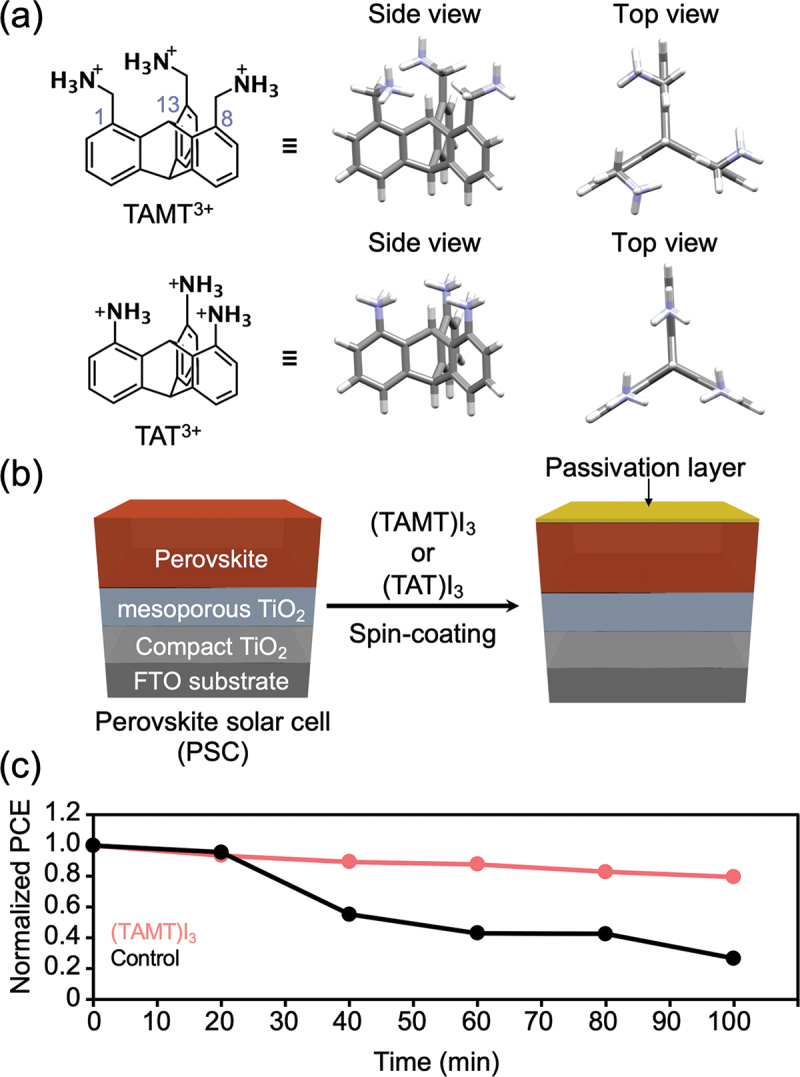
(a) Chemical and schematic molecular structures of TAMT^3+^ and TAT^3+^. Carbon numbering of triptycene is shown in the framework of TAMT^3+^. (b) Illustrations of the formation of a surface-passivation layer for a perovskite solar cell (PSC) using (TAMT)I_3_ and (TAT)I_3_. (c) Time-dependence of the normalized PCE of PSCs with/without a passivation layer of (TAMT)I_3_. The chemical composition of the perovskite layer of the PSC is MA_0.13_FA_0.87_PbI_2.61_Br_0.39_ (MA: methylammonium, FA: formamidinium). Reproduced from ref. [117].

1,8,13-Substituted triptycenes, referred to as triptycene tripods, have demonstrated an excellent ability to form a 2D assembled structure on various substrates. [115] Moreover, 1,8,13-trimercapto- and 1,8,13-trimercaptomethyltriptycenes can form self-assembled monolayers (SAMs) on Au(111) surfaces, featuring dense molecular packing, large-area uniformity, and upright molecular orientation. [116] Inspired by the 2D structuring ability of triptycene, researchers examined 1,8,13-tris(ammoniomethyl)triptycene triiodide [(TAMT)I_3_] and 1,8,13-triammoniotriptycene triiodide [(TAT)I_3_] as the component of passivation layers for a hybrid perovskite solar cell (Figure 22a). [117] An isopropyl alcohol solution of (TAMT)I_3_ or (TAT)I_3_ was coated onto a binary mixed-perovskite film composed of MA_0.13_FA_0.87_PbI_2.61_Br_0.39_ [MABr (or PbBr_2_):FAI (or PbI_2_) = 0.13:0.87] (Figure 22b). Interestingly, the (TAMT)I_3_ having methylene spacers was shown to function as a good passivation layer for a hybrid perovskite solar cell, leading to an improvement of both the power conversion efficiency (PCE) and long-term stability (Figure 22c). This report is a remarkable illustration of how the methodology of exospheric supra-ceramics can improve the performance of hybrid perovskites.

## 7. Summary and outlook

We reviewed a new class of inorganic materials named supra-ceramics, in which molecular species (molecular ions, metal complexes, clusters, etc.) are incorporated into inorganic solids. Depending on how the molecular species are incorporated, we define two categories of supra-ceramics: endospheric and exospheric types. The former contains molecular ions within inorganic crystal lattices, whereas the latter involves functional molecules such as complexes and clusters at specific positions on the surface of inorganic materials. As demonstrated by representative examples presented in this review, the incorporated molecular species create new degrees of freedom derived from their complexity and anisotropy, leading to novel functionalities that have not been previously observed in conventional inorganic – organic materials. Despite the fascinating features of supra-ceramics, many obstacles remain to the development of new supra-ceramics because of the difficulty in integrating inorganic and organic components with distinct properties. Specifically, new scientific concepts and improved or optimized technologies are indispensable for the synthesis and characterization of supra-ceramics.

For the establishment of supra-ceramics as a research area, the exploration of synthetic protocols for molecular-incorporated inorganic compounds (i.e. endospheric supra-ceramics) is highly desirable. Looking ahead in synthetic inorganic chemistry, we propose a promising method for the synthesis of new supra-ceramics. It is well-established that molecular-incorporated inorganic compounds frequently form when metal oxides are heated in specific reactive gas atmospheres. Common examples include carbonation (i.e. CO_3_^2−^ incorporation) and hydroxylation (i.e. OH^−^ incorporation). However, significant potential exists for the discovery of unprecedented (oxy)carbonates and (oxy)hydroxides if syntheses are conducted under unconventional conditions.

For conventional ceramics such as metal oxides and mixed-anion compounds, the rigid-sphere approximation model of monatomic ‘ionic radius’ can be used to predict the possible structures to a certain extent. However, the structure of endospheric supra-ceramics containing molecular ions cannot be designed on the basis of a simple parameter such as ionic radius. In addition, molecular ions have new degrees of freedom (e.g. rotation) not present in monoatomic ions; it can be inferred that the governing factors for stable structures in supra-ceramic materials will become even more complex. Given these circumstances, the use of materials informatics (MI) and artificial intelligence (AI) in conjunction with classical first-principles calculations will be particularly important in the future for highly predictive material design and efficient material exploration in relation to supra-ceramics.

From an analytical perspective, the sophistication of existing analytical methods is highly desirable. For example, RIXS is a powerful tool that enables the characterization of the dynamic behavior of molecular species in inorganic solids. However, because of the lack of brightness in the soft X-ray region and the fact that the astigmatism-controlling mirror and diffraction grating are not optimized, the exact bond order of oxygen molecules and its charge exchange with the surroundings have not yet been clarified. Using next-generation synchrotron radiation of NanoTerasu (Sendai, Japan), whose brightness in the soft X-ray region is more than 100 times greater than that of SPring-8, and by introducing astigmatism-controlled mirrors and diffraction gratings optimized for high-resolution RIXS, an unrivaled ultra-high-resolution RIXS measurement system for light elements will be constructed (Figure 6). This system will enable experimental observation of chemical states and anharmonic oscillator behavior of up to carbon species, which play a key role in molecular units, and will serve as an eye for searching for conditions to create high-performance supra-ceramic materials. Quantitative approaches to measuring target materials (such as single-molecular species on solid surfaces) that often face detection limits are desirable. The use of informatics methods, such as Bayesian measurement, may help reveal weak spectroscopic signals buried in noise with low signal-to-noise ratios and extract physical quantities that are difficult to measure directly.[118]

In terms of evaluating physical properties and functions, the concept of supra-ceramics (Figure 2) is considered particularly important. Regarding heterogeneous photocatalysis, the highly selective CO_2_–formic acid conversion ability shown experimentally for KGF-9 [89] and supported by theoretical calculations [90] suggests that semiconductive CPs or MOFs assembled from ligands that can provide surface – SH groups become highly selective photocatalysts for the conversion of CO_2_ to formic acid. On this basis, KGF-10 [92] and [Pb(ATAT)(OAc)]_*n*_ [58] have been identified as new photocatalysts. This is an example of using the concept of ‘reaction’. The concept of supra-ceramics is useful not only in obtaining desired properties and functions but also for identifying new properties and functions in existing materials. That is, many new and known materials can be the subject of research in the study of supra-ceramics. At the same time, materials containing molecular units are clearly not equal to supra-ceramics. Therefore, transformative research in materials science through supra-ceramics also requires a change in our view of materials.

Finally, we would like to make an important point from the viewpoint of inorganic chemistry. As seen in examples of solid cyanide complexes, [52–54] unexpected new developments have emerged through the interplay of coordination chemistry and solid-state chemistry, which are two important areas of inorganic chemistry. From the standpoint of solid-state chemists, new research developments can be expected by designing solid materials with a coordination chemistry perspective, and conversely, by introducing solid-state chemistry viewpoints from the perspective of coordination chemists. In fact, such interdisciplinary research between coordination chemistry and solid-state chemistry has made significant progress in some specific topics such as heterogeneous photocatalysis for CO_2_ reduction [25] and dye-sensitized solar cells. [119] Such interdisciplinary research is expected to expand further in the future.
